# Autophagy impairment: a crossroad between neurodegeneration and tauopathies

**DOI:** 10.1186/1741-7007-10-78

**Published:** 2012-09-21

**Authors:** Melissa Nassif, Claudio Hetz

**Affiliations:** 1 Biomedical Neuroscience Institute, Faculty of Medicine, University of Chile, Santiago, Chile; 2 Center for Molecular Studies of the Cell, Institute of Biomedical Sciences, University of Chile, Santiago, Chile; 3 Neurounion Biomedical Foundation, Santiago, Chile; 4 Department of Immunology and Infectious Diseases, Harvard School of Public Health, Boston, MA 02115, USA

## Abstract

Most neurodegenerative diseases involve the accumulation of misfolded proteins in the nervous system. Impairment of protein degradation pathways such as autophagy is emerging as a consistent and transversal pathological phenomenon in neurodegenerative diseases, including Alzheimer's, Huntington's, and Parkinson's disease. Genetic inactivation of autophagy in mice has demonstrated a key role of the pathway in maintaining protein homeostasis in the brain, triggering massive neuronal loss and the accumulation of abnormal protein inclusions. However, the mechanism underlying neurodegeneration due to autophagy impairment remains elusive. A paper in *Molecular Neurodegeneration *from Abeliovich's group now suggests a role for phosphorylation of Tau and the activation of glycogen synthase kinase 3β (GSK3β) in driving neurodegeneration in autophagy-deficient neurons. We discuss the implications of this study for understanding the factors driving neurofibrillary tangle formation in Alzheimer's disease and tauopathies.

See research article http://www.molecularneurodegeneration.com/content/7/1/48

## Commentary

The progressive accumulation of misfolded proteins in the brain is implicated in a vast group of human diseases, including the most prevalent neurodegenerative disorders such as Alzheimer's disease (AD), Parkinson's disease, and amyotrophic lateral sclerosis. These diseases are classified as protein misfolding disorders (PMDs) [1]. Despite representing an extensive and active field of research, in most cases the factors driving protein misfolding and neurodegeneration in the most common sporadic PMD cases remain poorly understood. Alteration in protein homeostasis networks is emerging as a factor driving neuronal dysfunction, where disruption in protein quality control mechanisms and protein clearance pathways may contribute to the accumulation of abnormally folded proteins in the brain.

**Figure 1:**
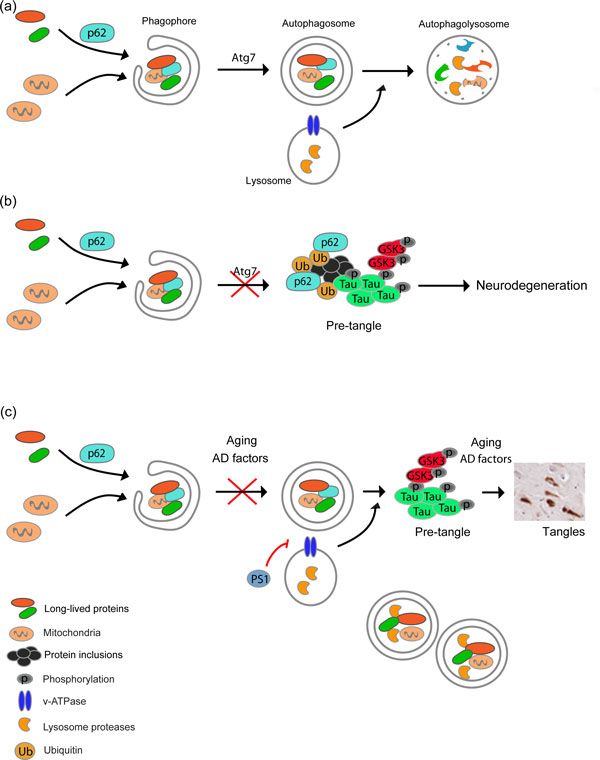
**Autophagy impairment and Alzheimer's disease**. **(a) **Schematic representation of the general steps of the autophagy pathway. **(b) **Ablation of the essential autophagy regulator Atg7 in neurons triggers spontaneous neurodegeneration, leading to the accumulation of ubiquitin-positive inclusions in a p62-dependent manner. Aggregate formation also involves the recruitment of partially phosphorylated Tau and active GSK-3β, resembling a 'pre-tangle' non-amyloidogenic state. **(c) **Speculative model: in Alzheimer's disease (AD), defects in the autophagy pathway due to genetic mutations, environmental factors and/or aging may contribute to the accumulation of abnormal protein aggregates and possible phospho-Tau on a pre-tangle state. For example, mutations in Presenilin-1 alter the pH of the lysosome, thus decreasing autophagy activity, which may enhance neurodegeneration and further accumulation of amyloid-β. Since during aging autophagy activity decreases over time, we speculate that autophagy impairment, together with other factors related to AD (genetic and environmental), may lead to a progressive flow from a pre-tangle to a neurofibrillary stage. This progression may be linked to the cognitive deficits associated with AD and tauopathies.

The macroautophagy pathway (here referred to as autophagy) is the main degradation route for long-lived proteins, damaged organelles, and protein aggregates. Autophagy is a highly regulated process, characterized by the formation of double- or multi-membrane vesicles (autophagosomes) that sequester portions of cytosol, which are then delivered for degradation following fusion with lysosomes (Figure 1a). Stimulation of autophagy results in the degradation of most aggregate-prone mutant proteins genetically linked to PMDs [2]. In addition, the accumulation of most of these mutant proteins upregulates autophagy activity through poorly defined mechanisms, and operates as a detoxification mechanism. This suggests that autophagy could be a good target for disease intervention; however, there is accumulating evidence that PMDs involve specific defects in particular steps of the autophagy process. Thus, manipulation of autophagy in neurodegenerative disease may have unpredictable outcomes, where further stimulation of the pathway may even overload the system with cargo proteins, accelerating disease.

AD is characterized by a massive accumulation of lysosomal-related vesicles in the degenerating neurons. Several studies have identified specific defects in the autophagy process in AD mouse models. For example, Ralph Nixon's group proposed that presenilin-1 (PS1) directly regulates the acidification of lysosomes by controlling the targeting of v-ATPase to these vesicles [3]. AD-linked PS1 mutations result in defective lysosomal proteolysis, having a detrimental impact on protein homeostasis and neuronal function [3]. In fact, genetic strategies to restore lysosomal function prevented neuropathology and cognitive deficits of an AD mouse model [4]. Remarkably, restoring lysosomal function in AD mice significantly decreased the accumulation of amyloid-β in the brain [4], placing autophagy impairment as a possible upstream component in the etiology of AD.

Accumulating evidence suggests that autophagy activity decreases during aging, which is the major risk factor for developing PMDs. In agreement with this idea, strategies that stimulate autophagy (such as rapamycin or resveratrol treatments, and caloric restriction) have potent anti-aging effects *in vivo *[2]. The first evidence indicating an important function of basal autophagy in the brain came from studies describing the effects of the genetic ablation of essential autophagy regulators such as *Atg5 *and *Atg7 *in mice [5,6]. Autophagy-deficient neurons undergo spontaneous degenerative changes that resemble those related to PMD, involving the accumulation of ubiquitin-positive inclusions, neuronal loss, motor dysfunction, and premature death [5,6]. Although it was initially assumed that the accumulation of protein inclusions was the trigger of neurodegeneration in those models, an elegant study demonstrated that ablating the adapter protein p62/SQSTM1 abrogated the accumulation of protein aggregates in *atg7 *deficient neurons, but did not prevent neuronal dysfunction [7]. Thus, the molecular mechanisms explaining the occurrence of neurodegeneration due to autophagy impairment remain an important open question.

In *Molecular Neurodegeneration*, Inoue and coworkers identified a surprising mechanism underlying degeneration in autophagy-deficient neurons, involving the engagement of a phospho-Tau-dependent pathway [8]. Accumulation of phosphorylated Tau in neurofibrillary tangles represents one of the major hallmarks of AD and other tauopathies, including frontotemporal dementia [9]. The authors generated two conditional *Atg7 *knockout mice focusing on the post-natal deletion in specific brain areas including hippocampal and glutamatergic neurons within the cerebral cortex, and dopaminergic neurons in the midbrain (*Dat-Atg7 *cKO). This strategy recapitulated previous findings obtained with developmental inactivation of *atg7 *in neurons, including autophagy impairment, neuronal loss, and accumulation of ubiquitin-positive inclusions. Due to increasing reports suggesting the occurrence of autophagy impairment in PMDs, the authors examined the levels of the most common proteins accumulated in these diseases. Unexpectedly, an evident redistribution of phospho-Tau into inclusions was detected in Atg7-deficient neurons, in addition to one of its kinases, glycogen synthase kinase 3β (GSK3β). In contrast, no accumulation of amyloid precursor protein (APP), TDP-43 or α-synuclein was observed (Figure 1b) [8]. Interestingly, a recent report also suggested that stimulation of mammalian target of rapamycin (mTOR)-independent autophagy can protect neurons against degeneration in mutant Tau transgenic mice due to its degradation of Tau aggregates [10], reinforcing the idea that autophagy also controls Tau levels. A systematic characterization of Tau phosphorylation sites in Atg7-deficient neurons indicated that the pattern observed was similar to an early 'pre-tangle' state that does not lead to formation of amyloid or fibrillar structures. Using a pharmacological approach to inhibit Tau phosphorylation or the genetic ablation of *tau *in mice, the authors partially reverted the neurodegenerative effects of Atg7 deficiency. Remarkably, the formation of ubiquitin/p62-positive inclusions remained intact after these manipulations, suggesting that Tau-mediated neurodegeneration may represent (i) a downstream pathological event of abnormal protein aggregation, or (ii) a parallel phenomenon triggered by an unknown mechanism. It will be interesting to determine if deletion of p62 in *Atg7 *knockout mice affects Tau/GSK3β redistribution and phosphorylation.

This study suggests for the first time a connection between autophagy impairment, Tau pathology, and neurodegeneration (Figure 1c), and given the emerging association between autophagy dysfunction and aging, provides clues about the possible cause of the most common sporadic forms of neurodegenerative diseases, offering an interesting possibility for future therapeutic intervention.

## References

[B1] Matus S Glimcher LH Hetz C Protein folding stress in neurodegenerative diseases: a glimpse into the ER Curr Opin Cell Biol 2011 23 239 252 10.1016/j.ceb.2011.01.003 21288706

[B2] Rubinsztein DC Mariño G Kroemer G Autophagy and aging Cell 2011 146 682 695 10.1016/j.cell.2011.07.030 21884931

[B3] Lee JH Yu WH Kumar A Lee S Mohan PS Peterhoff CM Wolfe DM Martinez-Vicente M Massey AC Sovak G Uchiyama Y Westaway D Cuervo AM Nixon RA Lysosomal proteolysis and autophagy require presenilin 1 and are disrupted by Alzheimer-related PS1 mutations Cell 2010 141 1146 1158 10.1016/j.cell.2010.05.008 20541250 PMC3647462

[B4] Yang DS Stavrides P Mohan PS Kaushik S Kumar A Ohno M Schmidt SD Wesson D Bandyopadhyay U Jiang Y Pawlik M Peterhoff CM Yang AJ Wilson DA St George-Hyslop P Westaway D Mathews PM Levy E Cuervo AM Nixon RA Reversal of autophagy dysfunction in the TgCRND8 mouse model of Alzheimer's disease ameliorates amyloid pathologies and memory deficits Brain 2011 134 258 277 10.1093/brain/awq341 21186265 PMC3009842

[B5] Hara T Nakamura K Matsui M Yamamoto A Nakahara Y Suzuki-Migishima R Yokoyama M Mishima K Saito I Okano H Mizushima N Suppression of basal autophagy in neural cells causes neurodegenerative disease in mice Nature 2006 441 885 889 10.1038/nature04724 16625204

[B6] Komatsu M Waguri S Chiba T Murata S Iwata J Tanida I Ueno T Koike M Uchiyama Y Kominami E Tanaka K Loss of autophagy in the central nervous system causes neurodegeneration in mice Nature 2006 441 880 884 10.1038/nature04723 16625205

[B7] Komatsu M Waguri S Koike M Sou YS Ueno T Hara T Mizushima N Iwata J Ezaki J Murata S Hamazaki J Nishito Y Iemura S Natsume T Yanagawa T Uwayama J Warabi E Yoshida H Ishii T Kobayashi A Yamamoto M Yue Z Uchiyama Y Kominami E Tanaka K Homeostatic levels of p62 control cytoplasmic inclusion body formation in autophagy-deficient mice Cell 2007 131 1149 1163 10.1016/j.cell.2007.10.035 18083104

[B8] Inoue K Rispoli J Kaphzan H Klann E Chen EI Kim J Komatsu M Abeliovich A Macroautophagy deficiency mediates age-dependent neurodegeneration through a phospho-tau pathway Mol Neurodegeneration 2012 7 48 10.1186/1750-1326-7-48 PMC3544596 22998728

[B9] Jucker M Walker LC Pathogenic protein seeding in Alzheimer disease and other neurodegenerative disorders Ann Neurol 2011 70 532 540 10.1002/ana.22615 22028219 PMC3203752

[B10] Schaeffer V Lavenir I Ozcelik S Tolnay M Winkler DT Goedert M Stimulation of autophagy reduces neurodegeneration in a mouse model of human tauopathy Brain 2012 135 2169 2177 10.1093/brain/aws143 22689910 PMC3381726

